# Ecofriendly first-derivative synchronous fluorometric method for simultaneous determination of atorvastatin and aspirin in pharmaceutical preparations

**DOI:** 10.1038/s41598-025-99718-x

**Published:** 2025-05-22

**Authors:** Hamed H. M. Abuseada, Osama I. Abdel Sattar, Ahmed W. Madkour, Ahmed S. Taha

**Affiliations:** Pharmaceutical Analytical Chemistry Department, Faculty of Pharmacy, Al-Azhar University, Cairo, 11751 Egypt

**Keywords:** Atorvastatin, Aspirin, Fluorimetry analysis, First derivative synchronous, Eco-friendly analysis, Chemistry, Physics

## Abstract

The combination of atorvastatin (ATO) and aspirin (ASP) is widely prescribed for preventing cardiovascular diseases, particularly in individuals at risk of atherosclerosis and myocardial infarction. This study aims to develop and validate an eco-friendly spectrofluorimetric method using the first-derivative synchronous fluorescence (FDSSF) technique (Δλ = 80 nm) to simultaneously determine ATO and ASP in combined pharmaceutical formulations. A simple, rapid, cost-effective, and interference-free FDSSF method was employed. Ethanol was used as a green solvent, with a scanning rate of 500 nm/min and Δλ of 80 nm. Distinct fluorescence peaks for ATO and ASP were observed at 384 nm and 365 nm, respectively. The method was optimized for parameters influencing fluorescence intensity, ensuring enhanced sensitivity and selectivity. The method demonstrated excellent linearity (0.4–6 μg/ml for ATO, 1–10 μg/ml for ASP) with high sensitivity, as indicated by low LOD (0.03 μg/ml for ATO, 0.342 μg/ml for ASP) and LOQ (0.248 μg/ml for ATO, 0.714 μg/ml for ASP). The method was successfully applied to commercial tablet formulations and synthetic mixtures, yielding results comparable to HPLC, confirming its high accuracy and reproducibility. Environmental Assessment: The method was evaluated for “greenness” (AGREE, Complex MOGAPI, NEMI), “whiteness” (RGB12), and “blueness” (BAGI), achieving high sustainability scores. The results confirmed its minimal environmental impact and eco-friendly nature. The FDSSF method provides a green, accurate, and reliable approach for simultaneously determining ATO and ASP in pharmaceuticals, offering high sensitivity, exceptional selectivity, and environmental sustainability.

**Supplementary Information:**

The online version contains supplementary material available at 10.1038/s41598-025-99718-x.

## Introduction

**Fig. 1:**
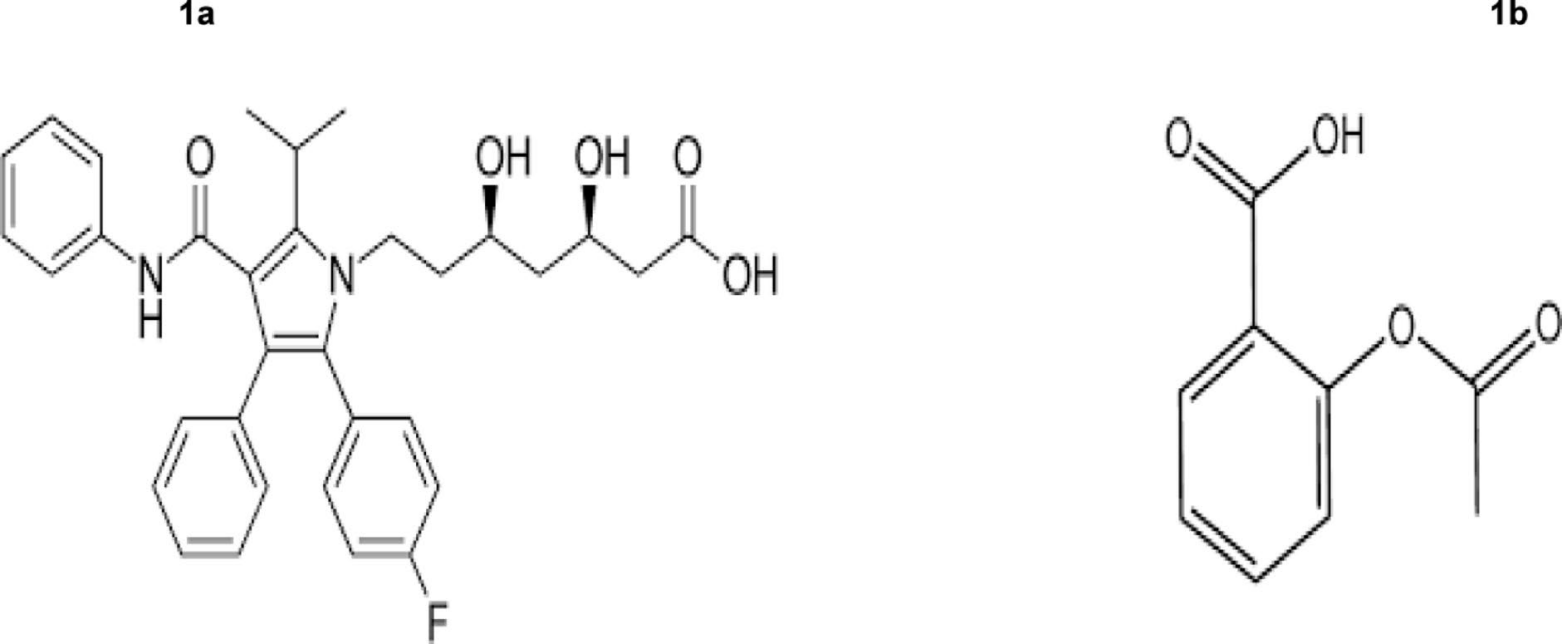
Shows the structural formulas for ATO (**1a**) and ASP (**1b**).

The quality and effectiveness of pharmaceutical formulations depend on precise and reliable analytical techniques. Ensuring drug safety and regulatory compliance requires robust methods for accurately quantifying active pharmaceutical ingredients (APIs) and detecting impurities that could compromise drug stability and patient health. Simultaneously quantifying multiple APIs remains a significant challenge, particularly in drug combinations like atorvastatin (ATO) (Fig. 1a) and aspirin (ASP) (Fig. 1b), which are widely used for cardiovascular disease prevention^1,2^.

Pharmaceutical analysis plays a crucial role in impurity profiling, which is essential for detecting trace levels of degradation products and contaminants. Traditional methods such as high-performance liquid chromatography (HPLC), spectrofluorimetry, spectroscopy, and capillary electrophoresis are commonly employed for impurity quantification. Integrating impurity profiling into analytical method development strengthens drug quality assessment and ensures adherence to regulatory standards. Recent advancements underscore the need for more sensitive and selective techniques to enhance drug purity and efficacy^3–7^.

This study aims to develop an accurate and efficient first-derivative synchronous fluorescence spectrofluorimetric (FDSFS) method for simultaneously determining ATO and ASP in bulk powders and pharmaceutical formulations.

Synchronous fluorescence spectroscopy (SFS) offers several advantages over conventional fluorescence techniques, including spectral band narrowing, simplified emission spectra, and enhanced selectivity^8^. These features make SFS highly effective for analyzing multi-component mixtures without requiring pre-separation steps. Combining SFS with derivative techniques further improves sensitivity and selectivity by reducing spectral overlap, a common limitation of traditional fluorescence methods. These advantages make it a promising approach for monitoring drug quality through trustworthy analytical techniques to ensure patient safety^9–13^. SFS and its derivatives have recently been utilized for identifying drug combinations in biological fluids and dosage forms^14–22^.

Despite the therapeutic significance of ATO-ASP combinations, most reported analytical techniques rely on HPLC, electrochemical, and spectrophotometric methods^23–26^. Conventional and synchronous spectrofluorimetric methods have yet to be utilized for analyzing the binary mixture of ASP and ATO in biological fluids or pharmaceutical dosage forms due to significant fluorescence spectral overlap. This limitation makes conventional fluorometric methods unsuitable for precise estimation. However, FDSFS effectively overcomes this obstacle by utilizing derivative amplitude measurements, which provide additional advantages in selectivity and sensitivity.

Fluorometric techniques offer significant advantages over simpler and cost-effective methods, such as spectrophotometry and electrochemical analysis. Unlike HPLC, which requires extensive sample preparation and costly solvents, spectrofluorimetric methods enable rapid analysis with minimal sample handling. Additionally, they achieve lower detection limits and higher specificity in complex pharmaceutical matrices, making them invaluable for routine quality control^27–33^.

As sustainability becomes a growing priority, analytical method development must align with the principles of Green Analytical Chemistry (GAC) to minimize environmental impact. GAC emphasizes reducing hazardous chemicals, limiting waste production, and optimizing energy efficiency throughout method development. The FDSFS method exemplifies this approach by significantly minimizing the use of hazardous solvents and reducing energy consumption, making it an environmentally friendly alternative to conventional techniques. To ensure its sustainability, standardized greenness assessment tools, including the Analytical Greenness Metric (AGREE), the Complex Multi-Objective Green Analytical Procedure Index (Complex MOGAPI), and the National Environmental Methods Index (NEMI), have been applied. These tools evaluate solvent selection, waste reduction, and energy efficiency, providing a comprehensive assessment of the method’s green profile. By integrating GAC principles and utilizing well-established assessment tools, the FDSFS method achieves a balance between analytical efficiency and environmental responsibility, paving the way for more sustainable practices in pharmaceutical and chemical analysis^34–41^.

A common challenge in pharmaceutical analysis is interference from excipients, essential components of drug formulations. Binders, fillers, and lubricants can affect fluorescence signals, potentially compromising sensitivity and specificity. However, the FDSFS method is meticulously optimized to eliminate any excipient interference. Through advanced spectral correction, precise solvent selection, and method refinement, it ensures fluorescence signals remain accurate and unaffected. By effectively addressing excipient interactions, this method guarantees reliable, interference-free drug quantification under all conditions^42^.

For any analytical technique to be widely adopted, it must demonstrate feasibility in real-world pharmaceutical applications. The FDSFS method has proven to be highly accurate and robust in laboratory studies. This refined technique ensures precise and dependable results while offering substantial practical benefits for the simultaneous analysis of ATO and ASP in combined pharmaceutical formulations. They present a cost-efficient, environmentally friendly, and sustainable solution for routine quality control in the pharmaceutical industry. With their straightforward implementation, minimal solvent usage, and operational efficiency, these methods are particularly well-suited for large-scale ATO and ASP formulation analysis. By enabling precise quantification while reducing environmental impact, they support regulatory compliance and advance sustainability in pharmaceutical testing^43–46^.

Emerging technologies are driving the evolution of pharmaceutical analysis. Miniaturized fluorescence sensors, quantum dot-based detection systems, and advanced spectroscopic techniques are gaining traction for drug quantification due to their superior automation, high throughput, and exceptional sensitivity. Integrating SFS with these cutting-edge innovations will further enhance its precision and applicability in pharmaceutical quality control, ensuring that modern analytical methods remain competitive and highly effective^6,47–51^.

By addressing key challenges in drug analysis, the proposed FDSFS method provides a selective, sensitive, and environmentally sustainable approach for the simultaneous determination of ATO and ASP. Its successful application in tablet formulations highlights its potential as a reliable tool for routine pharmaceutical quality monitoring, ensuring drug safety and efficacy.

## Experimental

### Materials

#### Pure samples

Pure ATO (99.73%) was supplied by Lipits Pharmaceuticals, 6th of October City, Egypt. Its purity was verified using the proposed method^52^.

Pure ASP (99.69%) was obtained from GlaxoSmithKline Pharmaceutical Industries, Cairo, Egypt, and its purity was verified using the established method^53^.

#### Pharmaceutical preparation

Atorlip Asp-20 tablets, manufactured by Cipla Ltd., India, were labeled to contain 20 mg of ATO and 75 mg of ASP. The tablets were procured from a local market in India.

#### Chemicals and reagents

The water used in the process was freshly distilled, and all reagents and solvents were of analytical grade.

- Organic solvents: Analytical-grade 1-propanol, ethanol (98.8%), acetonitrile, methanol, tetrahydrofuran, and chloroform were obtained from Sigma-Aldrich (Germany).
- Chemical reagents: Glacial acetic acid, boric acid, sodium hydroxide, sodium acetate, monobasic potassium phosphate, potassium chloride, Methylcellulose (MC, 0.5% aqueous solution), Tween 80 (0.5% aqueous solution), sodium dodecyl sulfate (SDS, 0.5% aqueous solution) and potassium bi-phthalate were supplied by El-Nasr Company (Egypt).
- Buffer solutions: Prepared according to the US Pharmacopeia guidelines:
- pH 2 buffer: Potassium chloride and hydrochloric acid.
- pH 3 buffer: Acid phthalate buffer.
- pH 4–5 buffer: Acetate buffer.
- pH 6–7 buffer: Phosphate buffer.
- pH 9–10 buffer: Alkaline borate buffer.

#### Apparatus

- All measurements were carried out using the Jasco FP-6200 Spectrofluorometer (Tokyo, Japan), with spectral data processed through Jasco Spectra Manager software. The monochromator slit widths were set to 10 nm, and a 1 cm quartz cell was used. Measurements were performed at medium sensitivity.
- A Jenway 3510 pH meter (England) with a reference electrode (model 924017 LO3-Q11C) was used for Ag/AgCl measurements.
- A Precisa 125A analytical balance (Switzerland) was employed for precise weighing.

#### Standard and working solutions

Stock standard solutions of ATO (1000 μg/mL) and ASP (1000 μg/mL) were prepared by dissolving 100 mg of each drug in 50 mL of ethanol (98.8%) in separate 100 mL volumetric flasks. The solutions were then diluted to the mark with the same solvent. To obtain the working solutions, 2.5 mL of each stock solution was transferred into a 100 mL volumetric flask and further diluted with ethanol to the final volume, yielding ATO and ASP concentrations of 25 μg/mL.

## Procedures

### Creating calibration graphs

Aliquots of the working standard ATO and ASP solutions (25 µg/mL) were transferred into 10-mL volumetric flasks. After adding 1 mL of 0.2 M acetate buffer (pH 5), the solutions were diluted to the 10 mL mark with ethanol and thoroughly mixed to ensure homogeneity. Synchronous fluorescence spectra were recorded by scanning both monochromators at a constant Δλ of 80 nm. The first derivative of each synchronous spectrum was obtained using a data interval of 15 points at 384 nm and 365 nm for ATO and ASP, respectively, and the corresponding peak amplitudes were measured. A blank experiment was conducted in parallel. Calibration curves for each drug were constructed by plotting the first derivative (1D) amplitude against the final concentration in µg/mL, followed by the derivation of the corresponding regression equations and calibration graphs.

### Procedure for laboratory-prepared mixtures

The selectivity of the proposed method for ATO in the presence of ASP was assessed by analyzing laboratory-prepared mixtures containing ATO and ASP in various ratios. Five distinct 10-mL volumetric flasks were prepared, each representing a different mixture ratio (1:3.75). For each mixture, appropriate volumes of ATO and ASP working solutions (25 μg/mL each) were transferred to achieve the desired concentrations and then diluted to the mark with ethanol. The procedure outlined under linearity and calibration graph preparation was followed. The concentrations of ATO and ASP in each mixture were determined using their respective calibration curves, and the percentage recovery (%R) was calculated to assess the method’s accuracy and selectivity.

### Pharmaceutical application

Ten Atorlip Asp tablets, each containing 20 mg of ATO and 75 mg of ASP, were accurately weighed and finely ground into powder. A portion equivalent to one tablet was transferred to a 100 mL volumetric flask containing 40 mL of ethanol. The mixture was subjected to vigorous shaking for 20 min, then filtered, and the volume was adjusted to 100 mL with ethanol. The resulting solution was further diluted with ethanol to obtain five distinct concentrations. The samples were analyzed according to the specified methods for linearity and calibration curves, and the percentage recovery of each drug was determined.

### Optimization of experimental conditions

The fluorescence intensity-affecting parameters of ATO and ASP were thoroughly examined and optimized. Some variables were individually adjusted, while others remained constant. These parameters include buffer volume, diluting solvent, pH, buffer type, and Δλ.

### Selection of optimum Δλ

The optimal Δλ value is critical for achieving peak sensitivity, resolution, and overall performance in the synchronous fluorescence scanning (SFS) method. This parameter directly impacts both the intensity of the synchronous signal and the spectral profile. To assess its effect, a broad Δλ range (20–120 nm) was examined for ATO and ASP. The results indicated that 80 nm was the optimal Δλ, producing two well-defined peaks with ideal spectral shapes. In contrast, lower Δλ values led to reduced fluorescence intensity, while excessively low values caused spectral distortion for both drugs.

### The impact of surfactants

Various surfactants were tested to determine their effect on fluorescence intensity. (Fig. 6a) summarizes the results for cetrimide, carboxymethylcellulose, sodium dodecyl sulfate (SDS), β-cyclodextrin, and Tween 80. Compared to the initial trials without surfactants, neither ASP nor ATO exhibited a significant enhancement in fluorescence intensity with any of the tested surfactants.

### pH and buffer effect

To assess the impact of pH on the fluorescence intensity of ASP and ATO (Fig. 6b), two buffer types were tested: 0.2 M acetate buffer (pH 4.5–5.6) and 0.2 M phosphate buffer (pH 6.0–7.0). The results revealed that ASP exhibited its highest fluorescence intensity and stability in mildly acidic conditions, with a peak at pH 5.0. However, at pH values above 5.0, spectral changes were observed, likely due to hydrolysis into salicylic acid, which can alter fluorescence properties.

Meanwhile, ATO demonstrated moderate fluorescence and solubility in slightly acidic to near-neutral conditions. Fluorescence quenching was observed at highly alkaline pH values (pH > 9), likely due to drug degradation. Based on these findings, 0.2 M acetate buffer (pH 5.0) was selected as the optimal medium, providing a balance between maximum fluorescence intensity, stability, and solubility for both ASP and ATO while minimizing hydrolysis and degradation^54–56^.

### Effect of diluting solvents

When ATO and ASP were diluted in various solvents, including ethanol, acetonitrile, methanol, n-propanol, and water (Fig. 6c), their fluorescence intensities were maximized in ethanol. Consequently, ethanol was chosen as the optimal solvent due to its ability to provide the highest fluorescence intensity while ensuring compound stability and compatibility with the analytical method.

Ethanol’s balanced polarity and reduced quenching effects enhance the fluorescence signals of both ATO and ASP, making it superior to other tested solvents. Additionally, ethanol stands out as the most environmentally friendly choice. Unlike acetonitrile and other organic solvents, ethanol is biodegradable, derived from renewable sources, and has a lower toxicity profile, making it safer for researchers and the environment. Its reduced environmental impact aligns with green analytical chemistry principles, promoting sustainability without compromising analytical performance. Thus, ethanol was selected as the preferred solvent for achieving maximum fluorescence intensity, chemical stability, and reproducibility while ensuring an eco-friendly approach in this study^54^.

## Findings and discussions

**Fig. 2:**
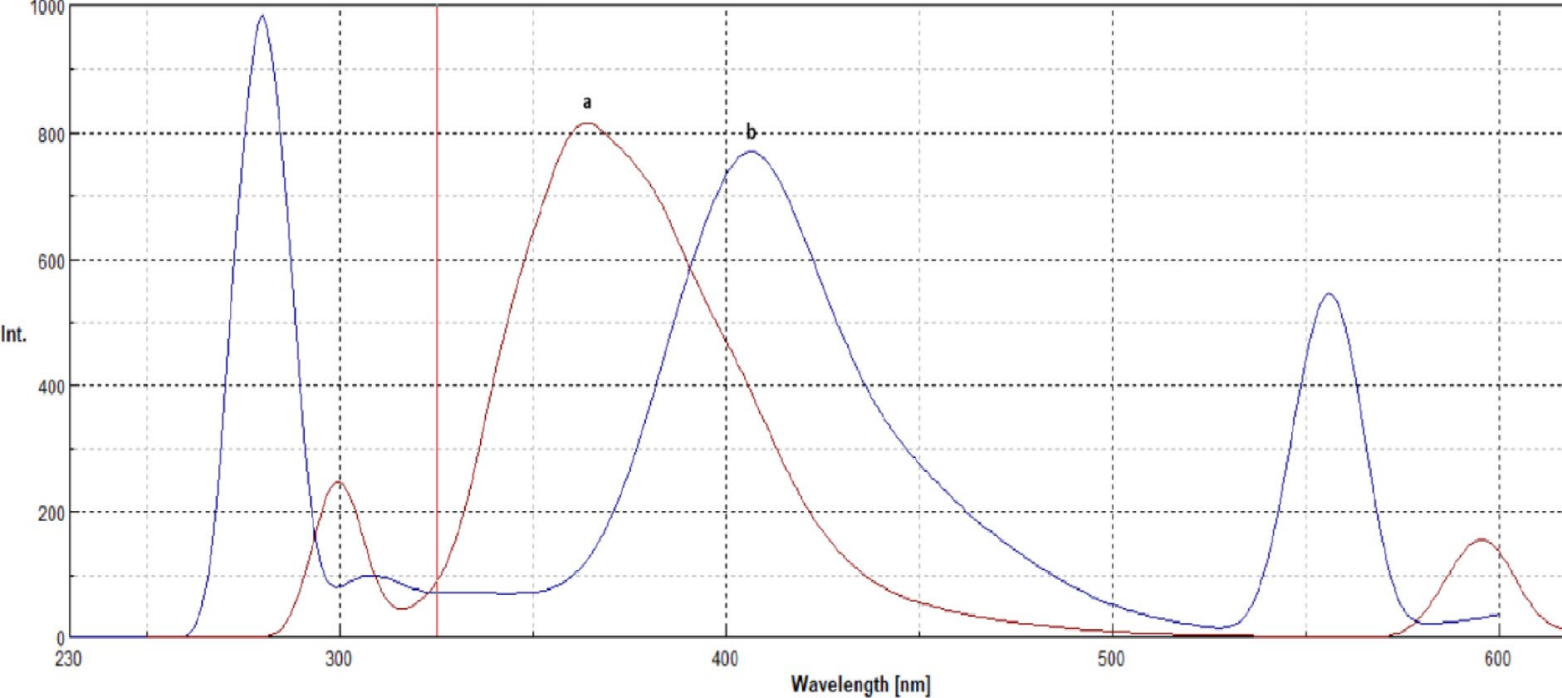
Shows 10 μg/ml of ATO, and 20 μg/ml of ASP overlapped emission fluorescence spectra.

In ethanol, Aspirin (ASP) and Atorvastatin (ATO) exhibit native fluorescence at 405 nm and 364 nm, respectively, when excited at 285 nm and 300 nm (Fig. 2). These excitation wavelengths were selected based on their intrinsic fluorescence properties, ensuring optimal excitation of each analyte to generate a strong and distinguishable emission signal. However, due to significant spectral overlap (Fig. 2), conventional fluorescence techniques lack sufficient selectivity for simultaneous determination.

**Fig. 3:**
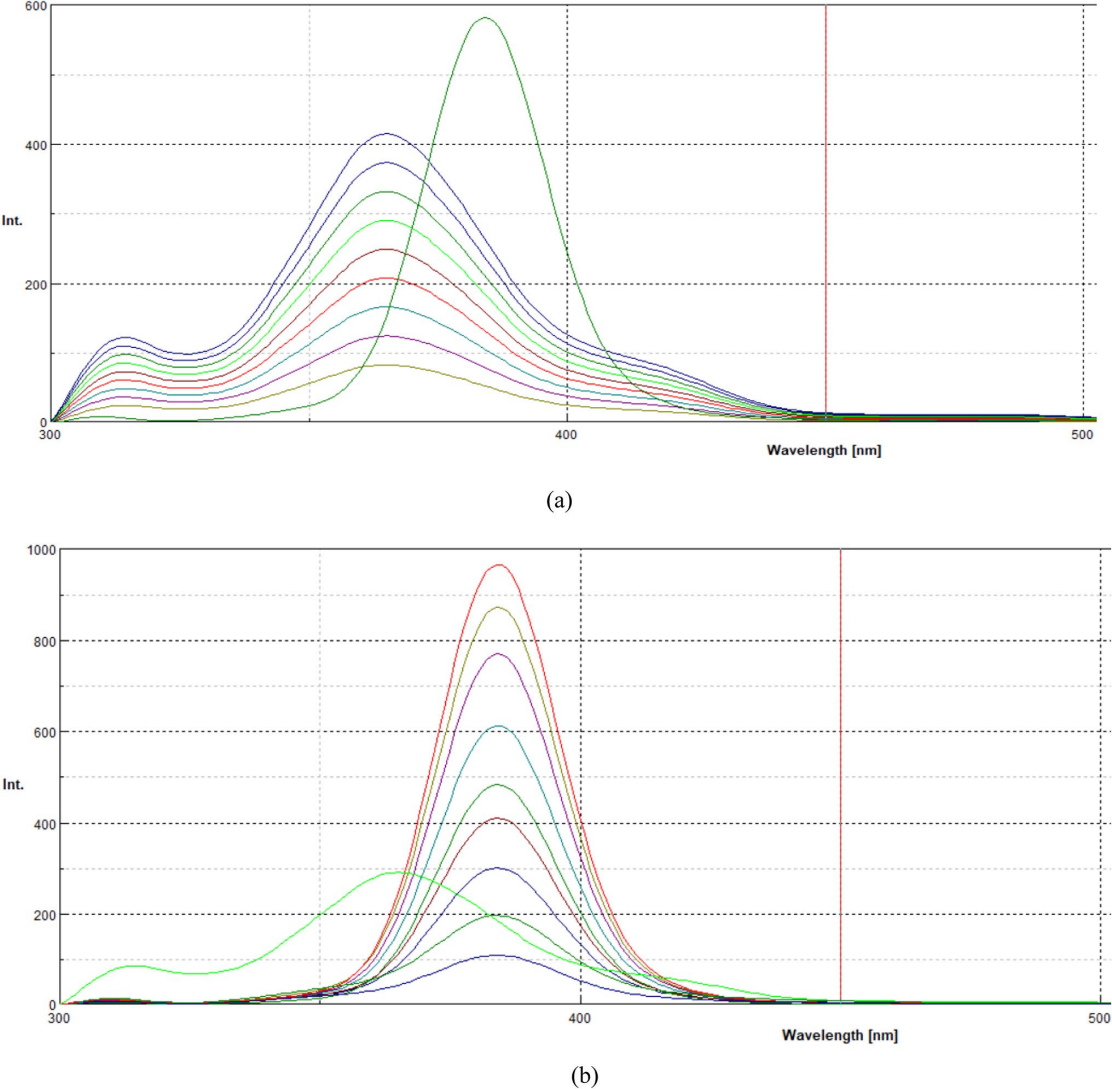
(**a**) Synchronous fluorescence spectra of ASP (8 μg/ml) and ATO (1, 1.5, 2,2.5,3, 3.5,4, 4.5, and 5 μg/ml). (**b**) Synchronous fluorescence spectra of ATO (2 μg/ml) and ASP (2, 3,4, 5,6,7,8,9 and 10 μg/ml).

To address this limitation, synchronous fluorescence spectroscopy (SFS) was employed to enhance selectivity and facilitate the differentiation of both drugs. Figure 3a shows the synchronous fluorescence spectra of ATO at varying ASP concentrations, while Fig. 3b illustrates the SFS spectra of ASP in the presence of different ATO concentrations. Despite applying SFS, considerable spectral overlap persisted, posing a challenge for independent quantification.

**Fig. 4:**
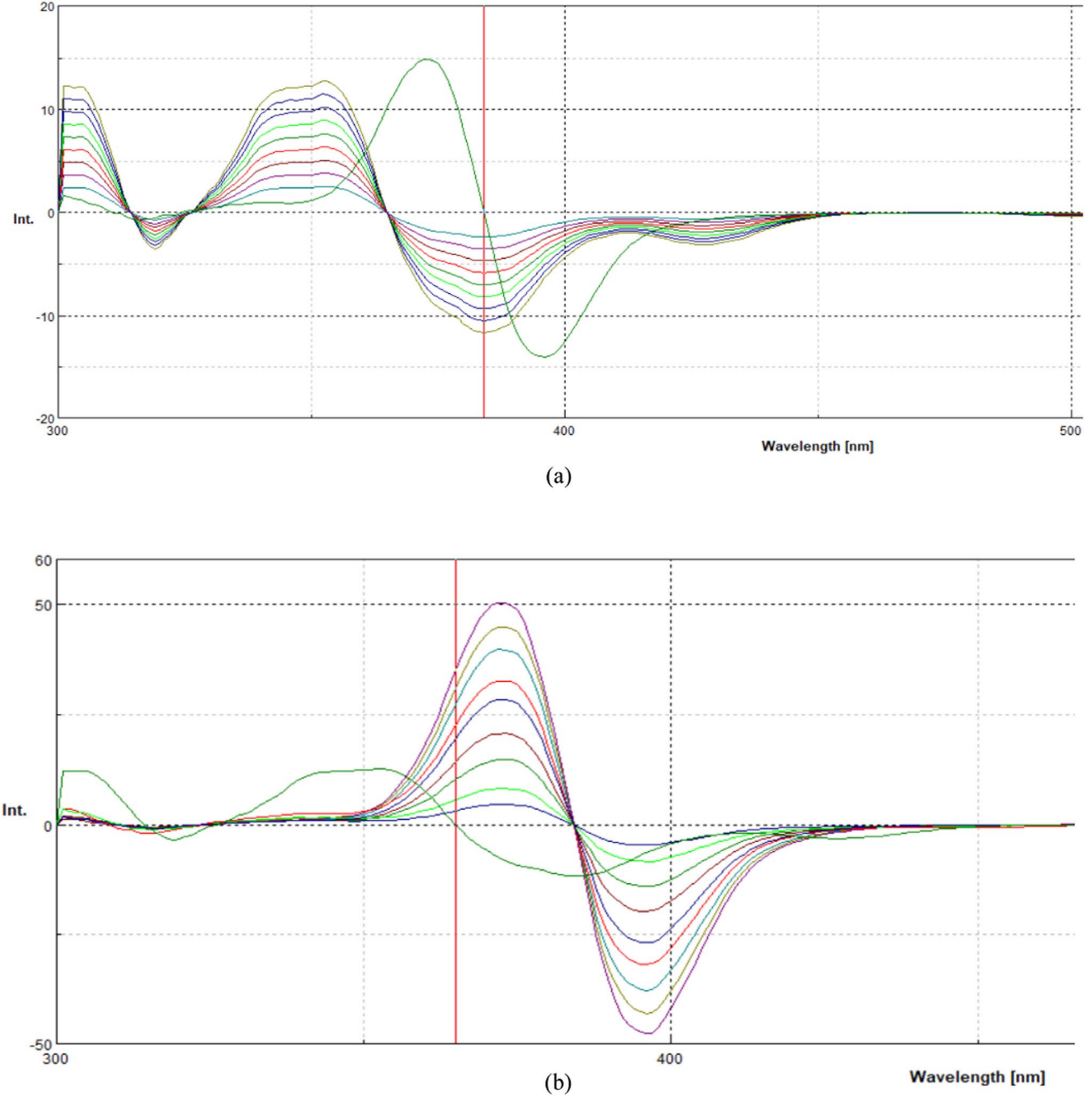
(**a**) First-derivative synchronous fluorescence spectra of ASP (8 μg/ml) and of ATO (1, 1.5, 2,2.5,3, 3.5,4, 4.5, and 5 μg/ml) at 384 nm. (**b**) First-derivative synchronous fluorescence spectra of: ATO (3 μg/ml) and of ASP (2,3,4,5,6,7,8,9 and 10 μg/ml) at 365 nm.

**Fig. 5:**
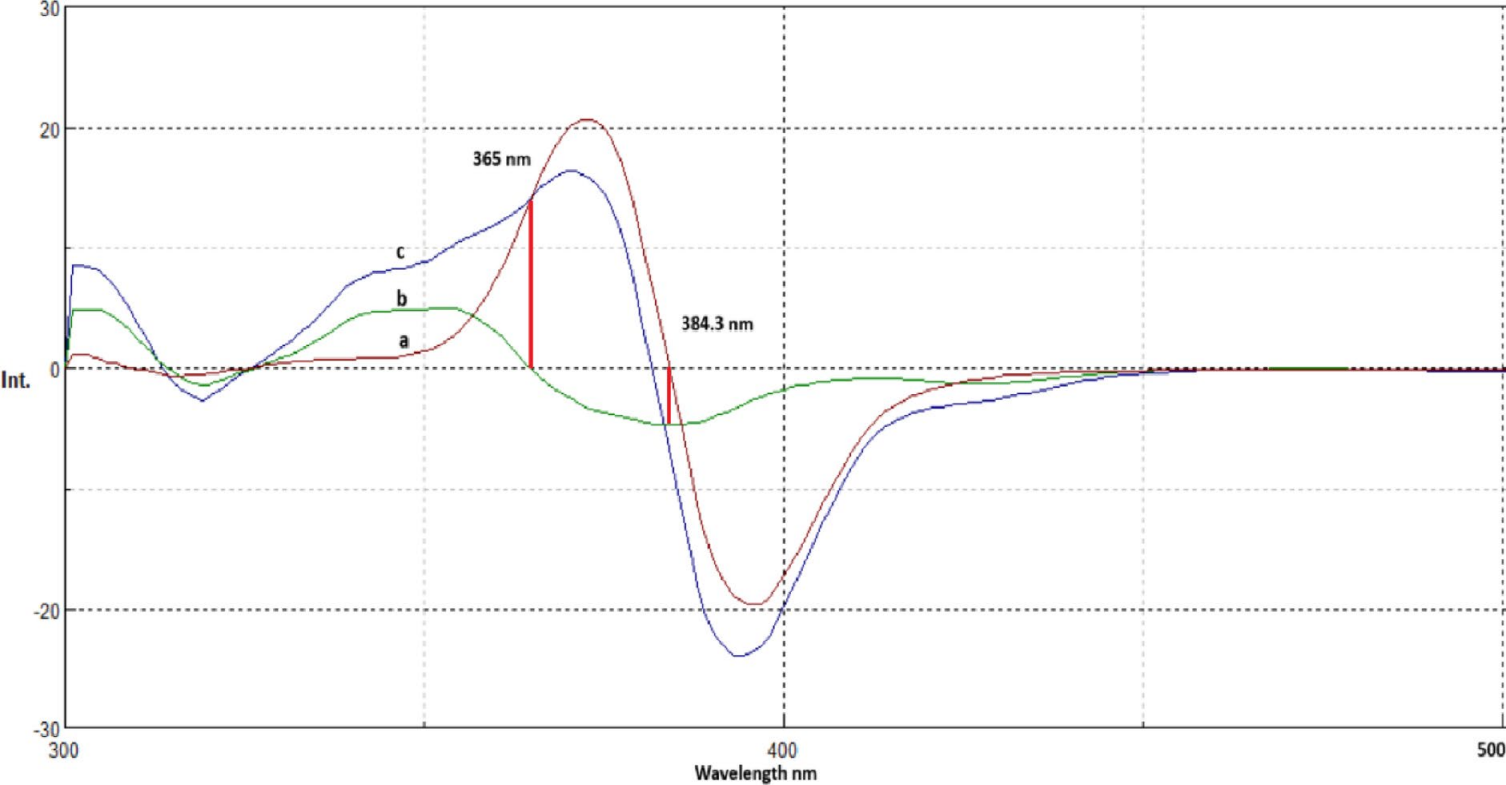
First-derivative synchronous fluorescence spectra of the prepared mixture. (**a**) ASP (6 μg/ml); (**b**) ATO (2 μg/ml); and (**c**) the mixture of 6 μg/ml ASP and 2 μg/ml ATO.

The first derivative synchronous fluorescence spectroscopy (FDSFS) was applied to enhance selectivity and eliminate spectral interference (Fig. 4a, b). Under optimized conditions (Fig. 5), ATO was accurately determined at 384 nm, while ASP was quantified at 365 nm. The selection of Δλ = 80 nm was systematically optimized to enhance spectral resolution and minimize peak overlap. This choice provided multiple analytical advantages, including suppression of background fluorescence, improved differentiation between ATO and ASP signals, and an enhanced signal-to-noise ratio, leading to superior fluorescence intensity and analytical precision. Furthermore, the FDSFS method eliminates the need for derivatization reagents, toxic solvents, and extensive sample preparation, making it a cost-effective, eco-friendly approach aligned with green analytical chemistry principles.

**Fig. 6:**
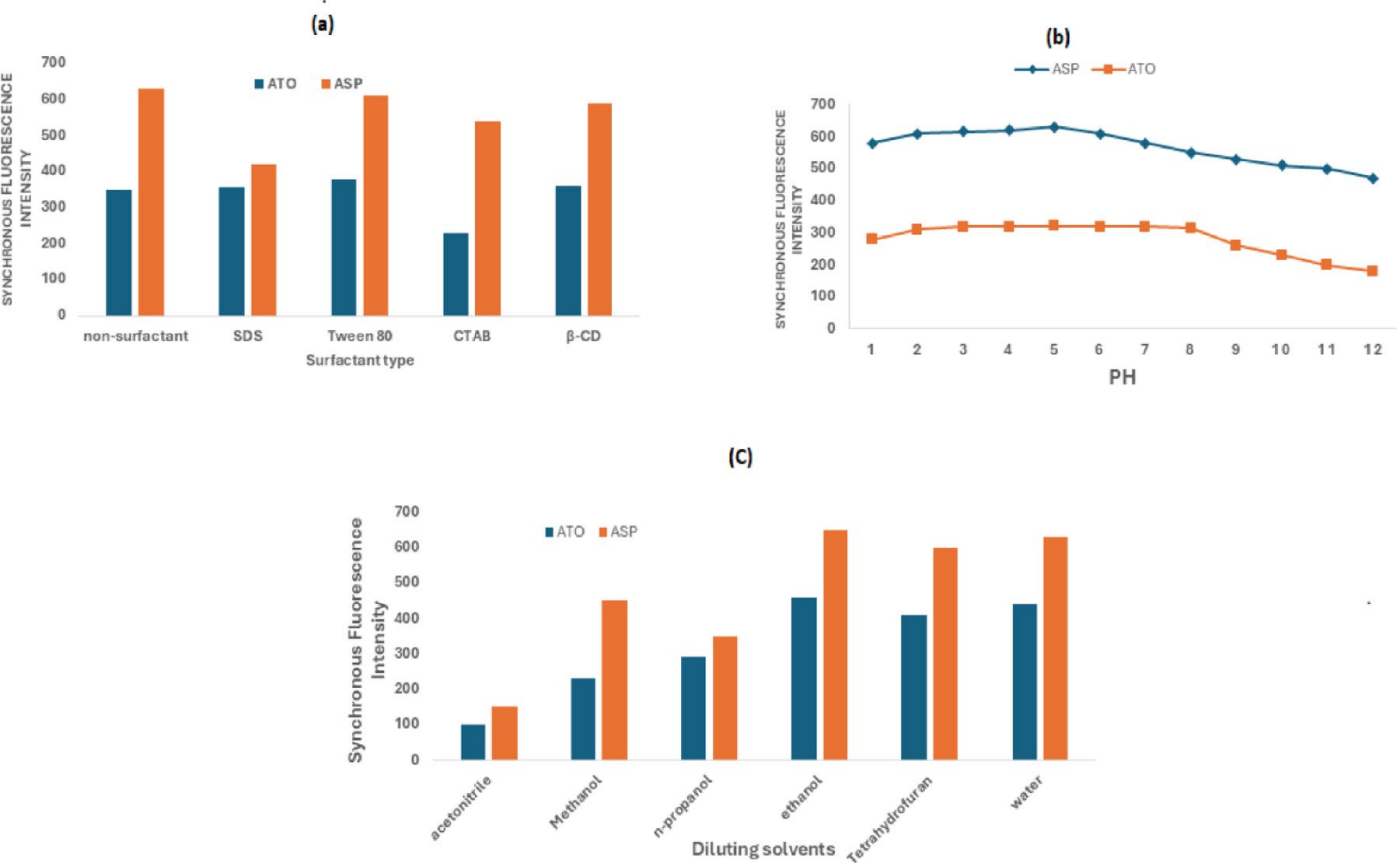
Optimization of method conditions using ATO (2 µg/mL) and ASP (6 µg/mL).

The validated FDSFS method offers a simple, efficient, and environmentally friendly alternative to conventional chromatographic techniques for the simultaneous determination of ASP and ATO in pharmaceutical formulations. By optimizing excitation wavelengths based on intrinsic fluorescence properties and selecting an appropriate Δλ, the method ensures high selectivity and sensitivity. Additionally, it eliminates complex separation steps, making it highly suitable for routine quality control analysis in pharmaceutical industries. This technique is directly applicable to the analysis of ASP-ATO combination drugs in commercial dosage forms, ensuring compliance with regulatory standards while maintaining high accuracy, precision, and reproducibility. Its adaptability and efficiency make it a valuable tool for laboratories seeking rapid and reliable pharmaceutical analysis (Fig. 6).

### Comprehensive greenness, whiteness, and blueness evaluation

The “greenness” assessment used the NEMI Figure S1, Complex MOGAPI Figure S2, and AGREE tools. At the same time, the “blueness” and the “whiteness” were assessed using the BAGI tool and RGB12 algorithm, respectively^33–37^. The proposed spectrofluorimetric approach acquired high greenness, blueness, and whiteness values, as shown in Table 5.

## Method validation

According to ICH guidelines^54^, the proposed method was successfully validated for accuracy, precision, selectivity, linearity, limit of detection (LOD), and limit of quantification (LOQ), ensuring its reliability and suitability for pharmaceutical analysis.

### Linearity and range

When the peak amplitude (1D) was plotted against drug concentration, a linear correlation was observed for ASP over 1–10 μg/mL at 365 nm and ATO over 0.4–6 μg/mL at 384 nm. Linear regression analysis of the data yielded the following equations:

$$\begin{aligned}^{{1}} {\text{D }} = & \, 0.{\text{2138C }} + { 3}.{9893 }\left( {{\text{r }} = \, 0.{9999}} \right){\text{ for ATO at 384 nm}} \\^{{1}} {\text{D }} = & \, 0.{48}0{\text{6C }} - \, 0.{9123 }\left( {{\text{r }} = \, 0.{9998}} \right){\text{ for ASP at 365 nm}}, \\ \end{aligned}$$

where ^1^D represents FDSFS peak amplitude, C is the drug concentration in μg/mL, and r is the correlation coefficient. This indicates that the calibration graphs are linear, as detailed in Table 1.

**Table 1:** Regression and validation data for the suggested FDSSF procedure’s assessment of ATO and ASP.

Parameter	ATO	ASP
Wavelength (nm)	384 nm	365 nm
Linearity range (μg/ml)	0.4–6 μg/ml	1–10 μg/ml
Determination coefficient (r^2^)	0.9998	0.9999
Intercept	3.9893	-0.9123
Slope	0.2138	0.4806
LOD (μg/mL)	0.031 μg/ml	0.342 μg/ml
LOQ (μg/mL)	0.248 μg/ml	0.714 μg/ml
Accuracy (%R)^a^	99.51	100.38
Precision (%RSD)^b^		
Repeatability	1.406	1.241
Intermediate precision	1.117	1.051
Robustness^c^ (%RSD)		
Δλ (± 1 nm)	1.2	1.356
pH (± 0.1)^c^	1.02	1.089
Phosphate buffer volume (± 0.1 ml)^c^	1.65	0.572

^a^Nine determinations on average (three concentrations, three times).

^b^The % RSD of nine measurements (three concentrations, three repetitions).

^c^%RSD of determination of three concentrations of each drug after slight changes in the pH (± 0.1) and phosphate buffer volume (± 0.1 ml).

### Limits of detection and quantitation

Following ICH guidelines, the LOD and LOQ were determined using the following formulas:

$${\text{LOD }} = { 3}.{3 }\sigma /{\text{S}}. {\text{LOQ }} = { 1}0 \, \sigma /{\text{S}}.$$

where S represents the slope of the calibration curve and σ is the standard deviation of the y-intercepts of the regression lines.

The low LOD and LOQ values obtained (see Table 1) confirm the high sensitivity of the method. Specifically, the detection limits for ASP and ATO were 0.342 μg/mL and 0.031 μg/mL, respectively. The quantification limits were 0.714 μg/mL for ASP and 0.248 μg/mL for ATO, demonstrating the method’s capability to detect and quantify the drugs at very low concentrations.

### Accuracy and precision

To ensure the reliability of the proposed method, recovery studies were conducted at 80%, 100%, and 120% of the assay concentration, following ICH guidelines. The accepted recovery range for accuracy is typically 98.0–102.0%. Accuracy, expressed as percent recovery (%R), was evaluated for ATO at 0.8, 1, and 1.2 μg/mL and ASP at 1.6, 2, and 2.4 μg/mL. The results, in Table 1, confirm the method’s accuracy and robustness, as all recovery values fell within the accepted range.

For precision assessment, three concentration levels within the linearity range—ATO (1, 2, and 3 μg/mL) and ASP (2, 4, and 6 μg/mL)—were analyzed. Repeatability was evaluated through triplicate measurements on the same day (intra-day precision), while intermediate precision (inter-day precision) was assessed over three consecutive days. Precision was expressed as percent relative standard deviation (%RSD), with an accepted RSD limit of ≤ 2.0% as per ICH guidelines. The low %RSD values, as detailed in Table 1, confirm the method’s high precision and reproducibility, remaining well within the accepted limit.

### Specificity and selectivity

The proposed technique was employed to confirm the method’s specificity. The determination of ATO in ASP-prepared laboratory mixtures demonstrated excellent selectivity, as presented in Table 2. Additionally, the standard addition method was applied to further assess specificity, with results detailed in Table 3.

**Table 2:** ASP and ATO assessment using the proposed technique in laboratory-prepared mixes.

Added ATO (μg/mL)	ASP found (μg/mL)	ATO (%R)	Added ASP (μg/mL)	ASP found (μg/mL)	ASP (%R)
0.5	0.504	100.77	1.875	1.857	99.05
1	0.981	98.12	3.75	3.744	99.83
1.5	1.499	99.91	5.625	5.552	98.7
2	2.027	101.35	7.5	7.451	99.34
2.5	2.521	100.83	9.375	9.508	101.42
Mean ± %RSD 100.196 ± 1.13612			Mean ± %RSD 99.668 ± 1.0632		

**Table 3:** ATO and ASP recovery investigation utilizing the proposed approach and standard addition procedure.

Drug	Pharmaceutical formulation taken (µg/ml)	Pharmaceutical formulation found^a^ (µg/ml)	Pure added (μg/ml)	Pure found^b^ (µg/ml)	Pure recovery (%R)
Atorvastatin	20	19.92	0.5	0.496	99.18
			1	0.986	98.57
			1.5	1.514	100.94
			2	1.974	98.72
	Mean ± %RSD				99.35 ± 1.097
Aspirin	75	75.21	1.875	1.899	101.28
			3.75	3.728	99.4
			5.625	5.662	100.65
			7.5	7.637	101.83
	Mean ± %RSD				100.79 ± 1.036

^a^Average of five assessments.

^b^Average of three assessments.

To evaluate the method’s applicability, ATO and ASP were quantified in Atorlip Asp tablets using the proposed approach. The results obtained through the standard addition method validated the accuracy of the analysis, showing a strong agreement with the labeled claim and confirming the absence of interference from excipients and additives.

A statistical comparison between the results of the proposed method and those obtained using a previously reported method^25^ demonstrated high accuracy and precision in assessing the studied drugs in their pharmaceutical dosage form. Furthermore, applying the T-test and F-test at a 95% confidence level (5) indicated no significant differences between the two methods, as shown in Table 4.

**Table 4:** Assessment of ATO and ASP in atorlip Asp tablets by the proposed approach and statistical comparison with the reported technique.

Parameters	Proposed method		Reported method^a^^12^	
	ATO	ASP	ATO	ASP
Number of measurements	5	5	5	5
Mean % recovery	99.83	99.8	100.48	99.85
% RSD	1.128	0.3	1.314	0.48
Variance	1.269	0.09	1.743	0.23
Student’s *T*-test^b^	0.828 (2.306)	1.545	–	–
*F*-value^b^	1.373 (6.388)	2.556	–	–

^a^Experiments number.

^b^Tabulated values of "t" and "F" at (*P* = 0.05) are shown in parenthesis.

### Robustness

The robustness of the proposed method was evaluated by making minor adjustments to the optimized conditions, including slight variations in pH (± 0.1) and phosphate buffer volume (± 0.1 mL) while maintaining all other parameters unchanged. Each factor was altered individually to assess its impact on method performance.

Notably, these small modifications did not significantly affect the fluorescence intensity, demonstrating the method’s reliability under slightly varied conditions. As shown in Table 1, the percentage RSD remained below 2%, confirming the robustness of the proposed approach.

## Conclusion

In this study, we developed an eco-friendly spectrofluorimetric method for the simultaneous quantification of ASP and ATO in combined pharmaceutical formulations. The method effectively resolves overlapping signals, ensuring high sensitivity and reliability in drug analysis.

Compared to conventional spectrophotometric and HPLC methods, this approach offers significant advantages, including enhanced sensitivity, simplicity, and the elimination of costly and complex instrumentation, making it particularly suitable for routine quality control in pharmaceutical laboratories.

Furthermore, the developed method demonstrated excellent selectivity and efficiency, providing accurate and precise results for ASP and ATO in tablet formulations. Its successful application to Atorlip Asp tablets underscores its practical utility in ensuring the safety and efficacy of combination drug products while promoting eco-friendly and cost-effective analytical practices.

The greenness of the proposed method was evaluated using three different assessment tools—AGREE, Complex MOGAPI, and NEMI, along with “whiteness” (RGB12) and “blueness” (BAGI), achieving high sustainability scores. These evaluations confirm that the developed method is environmentally friendly and superior to the reported HPLC method in terms of green analytical metrics, reinforcing its potential for sustainable pharmaceutical analysis, as shown in Table 5.

**Table 5:**
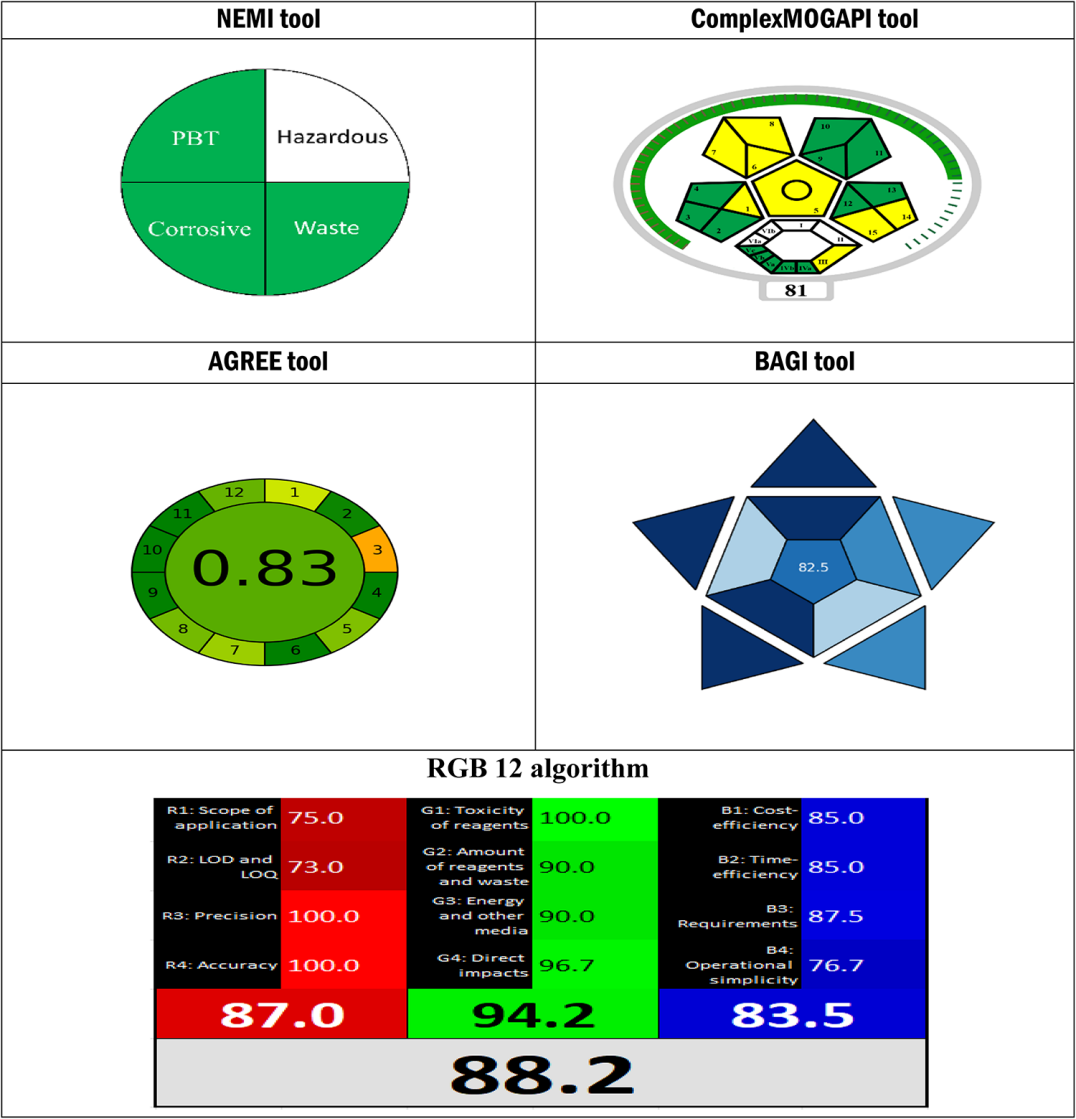
Comprehensive greenness, blueness, and whiteness evaluation of the proposed method.

## Electronic supplementary material

Below is the link to the electronic supplementary material.

Supplementary Material 1.

## Data Availability

The datasets used and/or analyzed during the current study are available from the corresponding author upon reasonable request.
