# Supplementary material for: Ecofriendly first-derivative synchronous fluorometric method for simultaneous determination of atorvastatin and aspirin in pharmaceutical preparations
Source: Sci Rep. 2025 May 22;15:17787. doi: 10.1038/s41598-025-99718-x (PMC12098671; doi:10.1038/s41598-025-99718-x)
Supplement: Supplementary file 1 — Supplementary Material 1. [file 41598_2025_99718_MOESM1_ESM.docx]

Comprehensive greenness, blueness, and whiteness evaluation:

Recent trends in analytical chemistry focus on incorporating sustainability concepts into method development. Evaluating the sustainability of analytical methods is crucial for understanding their environmental and economic impacts. However, sustainability is a multifaceted concept that encompasses greenness, waste minimization, safety, performance, and cost-effectiveness. No single assessment tool can comprehensively evaluate sustainability across all relevant parameters. Therefore, this work employs a multi-tool approach, combining complementary techniques to enable a holistic evaluation from different perspectives.

Greenness assessment was conducted using the National Environmental Method Index (NEMI), Complementary Green Analytical Procedure Index (Complex GAPI), and Analytical Greenness Metric (AGREE) tools. However, these greenness tools lack consideration for economic and performance factors. To address this limitation, the "blueness" aspect, which gauges Applicability and practicality based on analysis time, throughput, automation potential, and other applicability factors, was assessed using the Blue Applicability Grade Index (BAGI). Additionally, the concept of "whiteness" assessment was introduced through the Red-Green-Blue 12 (RGB12) algorithm. RGB12 evaluates analytical sustainability more comprehensively by incorporating critical parameters such as accuracy, cost, safety, and waste generation, providing a quantitative metric of overall sustainability.

In summary, this multi-tool approach provides a comprehensive and reliable assessment of sustainability by evaluating key parameters such as greenness, waste generation, safety, performance, practicality, and cost-effectiveness through qualitative and quantitative means. By employing multiple complementary tools, the limitations of individual approaches are overcome. The consistently high greenness, whiteness, and blueness scores demonstrate the sustainability merits of the proposed methods from diverse perspectives, validating their potential for implementation in sustainable analytical practices.

Evaluation of the method's greenness profile:

- NEMI tool:

NEMI tool provides a qualitative, simple, yet effective visual greenness assessment based on key parameters like toxicity, corrosiveness, and waste generation. NEMI leverages pictograms containing four key criteria, as shown in **Figure S1**, which are considered green if specific criteria are met. These criteria include: (1) chemicals employed in this approach not being classified as persistent, bio-accumulative, and toxic (PBT) as per the Toxic Release Inventory (TRI) and Agency Environmental Protection Agency's (EPA), (2) The chemicals employed in this procedure are not categorized as hazardous based on the criteria set forth by the (RCRA), specifically in terms of their classification under the U, P, F, D, or TRI lists of potentially hazardous waste, (3) The method's pH level is non-corrosive, falling within the range of 2 to 12, while (4) the amount of generated waste remains under 50 grams. In this work, NEMI pictograms were established, as shown in Table 5. The suggested method immediately stood out as a green method since three quadrants were colored green, satisfying four NEMI criteria. The pH of the methodology is 6.5, so it is non-corrosive as well, and the amount of waste that is produced is less than 50 g. Overall, while NEMI provides an initial broad screen of a method's general greenness, recent studies have highlighted some reliability concerns for performing greenness assessments using this tool alone due to the simplified pass/fail approach employed by NEMI based on a limited set of criteria. Therefore, in the current work, we apply NEMI as an initial first screen but combine it with more robust quantitative greenness metrics.

Figure (S1): Typical NEMI pictograms.


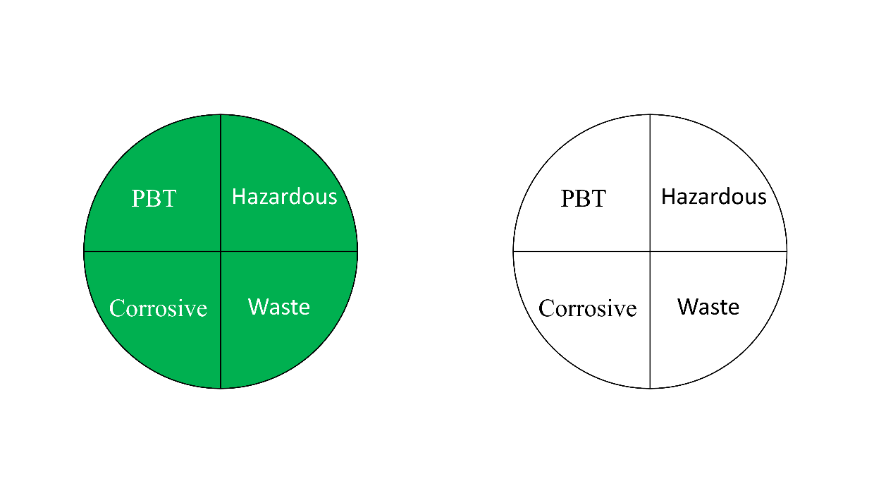


- ComplexGAPI tool:

The Complex MoGAPI enables a more detailed semi-quantitative greenness assessment. It builds upon the original MoGAPI framework by incorporating an additional hexagonal field for CHEM21 parameters, providing a more comprehensive evaluation of sustainability across all method stages, including sample collection, preservation, transportation, preparation, storage, and analysis. Complex MoGAPI employs a color-coded scale—from green to yellow to red—to effectively assess ecological impacts at each stage, as shown in Figure S2.

Critical quantitative metrics, such as the E-factor, are also calculated, with lower values indicating greater sustainability due to reduced waste generation. Additionally, Complex MoGAPI offers user-friendly software for generating pictograms, simplifying data visualization. In this study, Complex MoGAPI analysis demonstrated the exceptional greenness of the developed method, as reflected in the predominance of green indicators and the remarkably low E-factor, confirming its environmentally friendly profile and minimal waste production, as summarized in Table 5.

However, Complex MoGAPI primarily focuses on environmental aspects and does not fully address other sustainability dimensions, such as waste prevention, energy efficiency, and renewable materials. Therefore, integrating Complex MoGAPI with additional quantitative tools is recommended to achieve a more holistic sustainability assessment.

Figure (S2): The ComplexMoGAPI pictogram


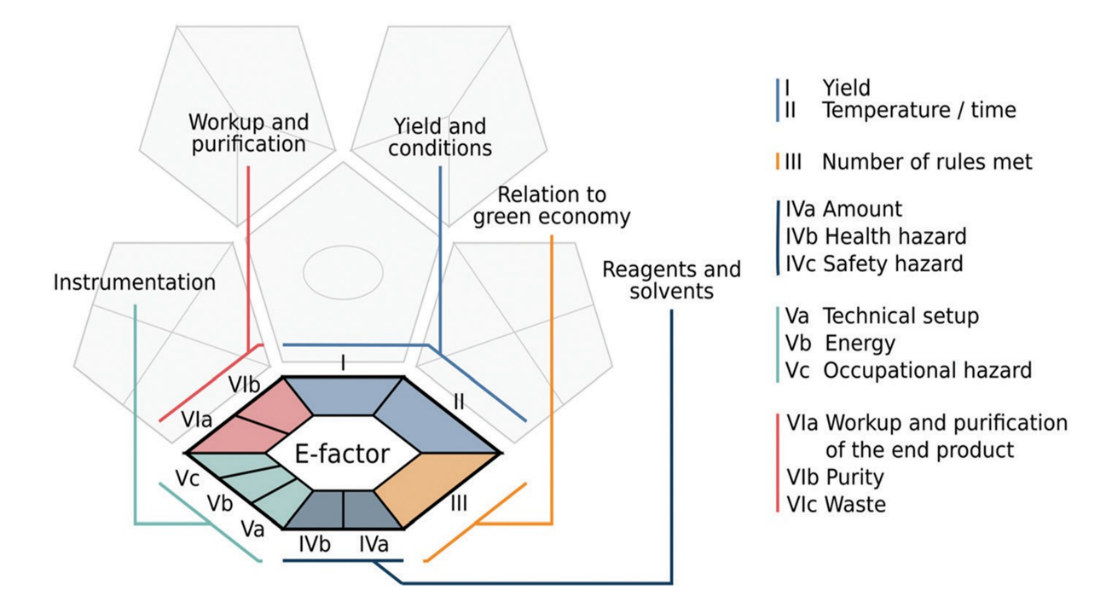


- AGREE tool:

AGREE metric provides another valuable quantitative approach for greenness assessment based on the 12 principles of green analytical chemistry (GAC). A key advantage of AGREE is the ability to assign custom weighting factors to parameters based on their relevance to the specific analytical method. This enables customized assessments focused on the most pertinent greenness criteria. The final AGREE score from 0 to 1 summarizes the overall method greenness, while the visual clock pictogram identifies areas needing improvement and can be conveniently applied using freely available software. In this work, an AGREE analysis for the suggested method was done. Based on the evaluation using AGREE, the suggested method exhibits outstanding greenness, achieving a high score (0.83), indicating its more efficient performance in terms of green principles. The graphs presented in Table 5 visually demonstrate the remarkable greenness of the suggested method, reinforcing its environmentally friendly characteristics and confirming its alignment with eco-friendly analytical practices. However, AGREE is limited to primarily environmental criteria related to green chemistry principles. Other key sustainability dimensions like safety, performance, and cost-effectiveness are not addressed. Thus, coupling AGREE with complementary tools assessing other sustainability dimensions is recommended for a more well-rounded assessment. Overall, AGREE is a valuable tool but should be part of a combined approach to guide the advancement of greener, more sustainable analytical techniques.

Assessment of the methods' blueness based on the BAGI tool:

While tools like NEMI, ComplexGAPI, and AGREE primarily focus on evaluating the greenness of analytical methods, the recently introduced BAGI specifically gauges the real-world functionality and applicability of these methods, a concept referred to as "blueness”. The BAGI tool enables a quantitative assessment of blueness across ten practical criteria, including analysis type, number of analytes, instrumentation requirements, sample throughput, sample preparation needs, analysis rate, reagents/materials used, preconcentration requirements, automation potential, and sample quantity. Each of these parameters is scored on a scale from 1 (worst) to 10 (best), and the composite BAGI index is computed as the geometric mean of these scores. In our study, we employed the BAGI tool to evaluate the blueness of our proposed method. Our method obtained impressive BAGI scores of 82.5, as presented in Table 5, indicating its excellent real-world applicability, high-throughput capabilities, potential for automation, and low operational costs. The BAGI assessment confirmed the outstanding blueness of our developed method, which translates to its practicality, functional efficiency, and real-world implementability. However, it is essential to recognize that BAGI specifically focuses on practical criteria and does not provide a holistic quantification of overall sustainability. To address this limitation, we additionally employed the RGB12 algorithm, which evaluates the composite analytical sustainability by considering greenness, performance, and practicality aspects. The consistently high scores obtained across these complementary tools verify the merit of our methods as implementable green chemistry alternatives, balancing environmental sustainability with practical applicability and analytical performance.

Assessment of the methods' whiteness based on the RGB12 algorithm:

In evaluating the whiteness profile of our proposed method, we introduced the RGB12, an effective tool for quantitatively assessing the overall sustainability level of analytical techniques. It comprises 12 distinct algorithms divided into three groups (red, green, and blue), each addressing crucial parameters related to sustainability. The green group (G1-G4) focuses on essential parameters such as toxicity, excessive waste and reagent utilization, energy consumption, and potential impacts on animals, humans, and genetically modified organisms. The red group (R1-R4) assesses validation criteria, including the method's applicability, limits of detection and quantification, precision, and accuracy. The blue group (B1-B4) evaluates factors related to affordability, time efficiency, practical considerations, and financial prerequisites. By applying the RGB12, the whiteness value, which represents the method's compliance with the principles of white analytical chemistry, is estimated by combining the scores obtained for each of the three color groups. As shown in Table 5, our suggested method attained remarkable whiteness values of 88.2. By employing RGB12 alongside other assessment metrics, we achieved a comprehensive, robust evaluation of sustainability, avoiding the problems inherent in any single approach. This systems-thinking approach, which utilizes multiple tools complementing each other, represents the best practice for conducting a thorough, unbiased assessment of the sustainability of analytical methods.
